# Exploring high dental anxiety subtypes using cluster analysis approach: a cross-sectional study

**DOI:** 10.1186/s12903-025-06820-7

**Published:** 2025-10-01

**Authors:** Mika Kajita, Vesa Pohjola, Eeva-Leena Kataja, Satu Lahti

**Affiliations:** 1 Department of Community Dentistry, Institute of Dentistry, University of Turku, 20014 Turku, Finland; 2 Systemic Approaches to Improve Cardiometabolic and Brain Health during Lifespan (SYS-LIFE), Co-funded by University of Turku and the European Union, Turku, Finland; 3 Section of Anesthesiology, Department of Diagnostics and General Care, Fukuoka Dental College, Fukuoka, Japan; 4 Research Unit of Population Health, Faculty of Medicine, University of Oulu, Oulu, Finland; 5 Department of Clinical Medicine, Turku Brain and Mind Center, FinnBrain Birth Cohort Study, University of Turku, Turku, Finland; 6 Centre for Population Health Research, University of Turku, Turku, Finland

**Keywords:** Dental anxiety, Cluster analysis, Pain perception, Sensory thresholds, Behavioral symptoms

## Abstract

**Background:**

Dental anxiety (DA) arises from exogenous learning experiences and endogenous vulnerabilities, yet little is known about how these factors combine within highly anxious adults. The aim was to identify empirical subtypes of high DA based on endo/exogenous factors and compare them according to self-reported oral health, care-seeking behavior, and preferred dental treatment conditions.

**Methods:**

A cross-sectional online survey of 399 Japanese adults (mean ± S.D. age = 43.5 ± 13.1 years; 50% women) who rated “very frightening” on the Japanese Single Dental Anxiety Question. Seven predictors were entered into a two-step cluster analysis: four exogenous variables (distressing dental and medical experiences, family DA, exposure to frightening dentists in the media) and three endogenous variables (fear of pain, pain catastrophizing, sensory-processing sensitivity). Demographics, Seattle DA classification, dental attendance pattern, self-reported oral health, and preferred treatment conditions (cognitive-behavioral therapy, conscious sedation, or general anesthesia) were compared between clusters using chi-square and Mann–Whitney U tests.

**Results:**

The optimal solution consisted of two clusters (silhouette = 0.30, maximum/ minimum ratio = 1.31). All participants in Cluster 1 (*n* = 173) had experienced distressing dental treatment and showed higher pain catastrophizing (*p* < 0.001). No one in Cluster 2 (*n* = 226) reported traumatic dental events and had lower catastrophizing, fewer regular check-ups (12% vs. 24%), a higher proportion who had never visited a dentist (14% vs. 0%), and less interest in DA treatment compared to Cluster 1.

**Conclusions:**

Two partially overlapping DA phenotypes emerged: C1 as “Trauma & Catastrophizing Compounded C1/ Still-Considers” and C2 as “Latent-Trait Driven C2/ Extreme Avoiders.” Tailored strategies that combine trauma-informed exposure for the former and sensory-adapted engagement for the latter may enhance the uptake of oral healthcare. Broader endogenous indicators should be added in future work to refine subtype boundaries.

**Supplementary Information:**

The online version contains supplementary material available at 10.1186/s12903-025-06820-7.

## Introduction

Dental anxiety is common and leads to avoidance of dental care and poor oral health [1, 2]. Dental anxiety is also associated with low oral health-related quality of life in adults [3–5] and also in children [6, 7]. Poor oral health, in turn, is connected to systematic diseases such as diabetes and cardiovascular disease [8, 9]. Accordingly, optimizing evidence-based interventions for dental anxiety should become a public health priority.

Conceptually, dental anxiety can be framed along a three-tier continuum: state anxiety, describing situational fear during treatment; elevated dental trait anxiety, a chronic pattern linked to previous negative dental experiences; and dental phobia, an extreme variant that meets DSM-5-TR criteria for specific phobia and causes functional impairment [10]. Recent meta-analytic evidence underscored that these phenomena overlap rather than form distinct categories and that treatment effects may vary across the continuum [10]. High dental anxiety, in particular, affects approximately 10–15% of the general population and constitutes a major driver of dental avoidance and oral disease burden [11]. Therefore, it represents a primary target for population-level prevention and early intervention strategies.

The etiology of dental anxiety is complex and multifactorial [12]. In line with prior literature [12, 13], two main origins for dental anxiety development can be conceptualized: exogenous and endogenous factors. Exogenous factors include learning from one’s own negative dental or medical experiences and indirect learning from, e.g., family members or the media [12]. Individual differences related to susceptibility to fear conditioning or constitutional vulnerability [14] and cognitive vulnerability (i.e., perceptions of specific situations) have also been reported. They are referred to as endogenous factors [12, 15]. Endogenous factors of dental anxiety also include, for example, complex biological predispositions, temperament, cognitive distortions, and pain-related factors. Fear of pain has been found to be an important endogenous component of dental anxiety [16, 17]. In addition to pain-related factors, hypersensitivity to sensory stimuli such as sound and light is another endogenous factor associated with dental anxiety [18]. Pain catastrophizing is one of the cognitive factors that make dental treatment scarier than it is [19]. A previous study by Ogawa (Kajita) et al. reported that sensory sensitivity and pain catastrophizing mediated the association between alexithymia, which refers to a tendency to have difficulty recognizing or describing one’s own emotions, and dental anxiety [20].

Several taxonomies have been proposed to describe sub-types of dental anxiety. One of them is the Seattle dental anxiety (DA) classification, comprising four categories: fear of specific stimuli, somatic-related anxiety, general nervousness, and distrust of dental professionals [21]. However, later population-based studies have reported that some individuals with high dental anxiety do not fit neatly into any of these categories or exhibit features from multiple types simultaneously [22]. More recently, van Houtem et al. used a twin cohort (*N* > 11,000) to confirm a three-factor structure—pain/invasive procedures, loss of control, and somatic sensations—although these factors frequently co-occurred in the same individual [23]. Another recent paper discussed two constructs of dental anxiety i.e., anticipatory and treatment-related dental anxiety [24]. These frameworks, although useful in identifying fear types, do not indicate which patients exhibit which pattern of dental anxiety nor which management approach would be most appropriate for each pattern.

Identifying patient clusters per se is clinically valuable because different etiological constellations imply different chair-side strategies. For example, a patient whose anxiety is driven mainly by conditioned trauma may benefit more from graded exposure and cognitive restructuring. In contrast, a patient whose anxiety stems from sensory hypersensitivity requires environmental adaptations (noise-canceling headphones, tinted lenses, weighted blanket) rather than exposure alone. Similar cluster-to-treatment mappings have already improved care in heterogeneous conditions such as depression [25] and chronic pain [26], where psychologically defined clusters predicted prognosis and led to subgroup-specific interventions.

Despite the recognized heterogeneity of high dental anxiety no study has yet applied cluster analysis to derive empirical, person-centered subtypes and relate them to dental attendance behavior and preferred dental treatment conditions. Therefore, pursued two aims. First, adults with high dental anxiety were clustered using seven exogenous (learning/experiential) and endogenous (psychological/biological vulnerability) variables. Second, the resulting clusters were compared based on demographics, the Seattle DA classification, self-rated oral health, dental attendance patterns, and interest in three specific dental treatment conditions: cognitive-behavioral therapy (CBT), conscious sedation, and general anesthesia. To assess robustness, a sensitivity analysis was performed by repeating the clustering with the Seattle DA classification alone.

## Methods

### Design and sample

This cross-sectional study was approved by the Ethics Committee of Fukuoka Dental College (No. 627) in Japan in 2023 and was conducted in accordance with the Declaration of Helsinki. The online survey was conducted using registrants of MSS Inc., a private Japanese online research company. These registrants are Japanese residents who voluntarily sign up to participate in online surveys and provide demographic information upon registration. Participants receive point-based compensation from the company in exchange for their cooperation. Further details about the panel management system are proprietary to the company.

At the time of the study, the overall panel composition of MSS Inc. included a wide range of occupational backgrounds. Among the 1.84 million active registrants, 35% were full-time company employees, 17% were part-time or student workers, and 9% were full-time homemakers. The panel also included students (8%), unemployed individuals (8%), self-employed individuals (4%), and public sector employees such as teachers and civil servants (5%). This diversity enhances the generalizability of survey data within the working-age population in Japan. The company monitors response quality by removing inconsistent responders and inactive users and by providing educational feedback to encourage reliable answers.

A stratified random sample of individuals aged 20 and over was selected from the panel, with a pre-agreed allocation of 400 participants balanced across gender and two age groups (20–50 and 51–80 years). These panelists had previously consented to receive invitations to participate in research surveys. An email invitation titled “Survey on Medical Topics” was sent to them, clearly stating that participation was voluntary. Before answering any screening questions, participants were shown an online explanation of the study’s purpose and asked to provide informed consent by clicking a confirmation button. Only those who gave consent were allowed to proceed.

As a screening item, participants answered the Japanese version of the Single Dental Anxiety Question (SDAQ), which asked, “Do you think that visiting a dentist is,” followed by a 5-point Likert scale ranging from “Not Frightening at all” to “Very Frightening” [27, 28]. Only those who selected “Very Frightening” were invited to the main survey. Those who selected lower options were excluded at this point.

### Questionnaire

The main survey consisted of validated psychometric scales and self-reported items designed to assess sociodemographic factors, dental anxiety, pain sensitivity, catastrophizing, sensory processing sensitivity, and prior experiences.

All participants were asked for information on sociodemographic characteristics (e.g., age, gender, occupation, and educational level), dental attendance pattern (divided into three categories: regularly, irregularly, and never), and self-reported oral health (divided into five categories: very poor, poor, average, good, and very good).

#### Dental anxiety

The Japanese version of the Modified Dental Anxiety Scale (MDAS) [29–31] was used to assess dental anxiety. MDAS is a five-item scale that assesses dental anxiety in five situations: going for treatment tomorrow, sitting in the waiting room, having one’s tooth drilled, having one’s teeth scaled and polished, and receiving local anesthetic injection. The questionnaire has high validity and reliability [29–31]. Responses were recorded on a 5-point Likert-type scale ranging from “not anxious” to “extremely anxious,” and the scores were summed to give a total score (range 5–25). Higher scores indicate greater dental anxiety. The two factors of the MDAS established in previous studies [32, 33], and suggested capturing dental anxiety originating from exogenous and endogenous sources [32, 33], were calculated as anticipatory dental anxiety (items 1 and 2; range = 2–10) and treatment dental anxiety (items 3–5; range = 3–15). The mean score for the Japanese MDAS, based on previous data, was 10.9, with a standard deviation of 3.8 [34]. The Cronbach’s alphas for the dimensions in this study were 0.883 and 0.821 for anticipatory dental anxiety and treatment dental anxiety, respectively.

#### Fear of pain

The Japanese version of the Fear of Pain Questionnaire–9 (FPQ–9) assessed fear of painful experiences encountered in everyday life and healthcare settings [34–36]. The questionnaire consists of 9 items, each item assessed on a five-point Likert scale, ranging from ‘not at all’ to ‘extreme.’ The overall score ranges from 9 to 45. Three subscales (severe, minor, and medical pain) exist. The questionnaire has high validity and reliability [34–36]. The mean score for the Japanese was 28.7, with a standard deviation of 7.4 from a previous study [34]. The Cronbach’s alphas for the dimensions in this study were 0.875, 0.814, and 0.809 for severe, minor, and medical pain, respectively.

#### Pain catastrophizing

The Japanese version of the Pain Catastrophizing Scale (PCS), a 13-item self-report measure, was used to assess three components of catastrophizing: rumination, magnification, and helplessness [37, 38]. The questionnaire has high validity and reliability [37, 38]. Responses were measured with a five-point Likert scale ranging from “strongly disagree” to “strongly agree.” Total scores range from 13 to 65. The mean score for the Japanese was 36.1, with a standard deviation of 11.2 [34]. The Cronbach’s alphas for the dimensions in this study were 0.898, 0.775, and 0.883 for rumination, magnification, and helplessness, respectively.

#### Sensory sensitivity

The Japanese Version of the Highly Sensitive Person Scale (HSPS)10-Item was used to measure individual differences in environmental sensitivity [39, 40]. The questionnaire has high validity and reliability [39, 40]. Responses were measured with a 7-level response format from “strongly disagree” to “strongly agree.” The scale assesses three components of sensory-processing sensitivity: ease of excitation, aesthetic sensitivity, and low sensory threshold. The mean of all items was used in this study. The mean score for the Japanese was 4.1, with a standard deviation of 1.1 [40]. The Cronbach’s alphas for the dimensions in this study were 0.892, 0.860, and 0.825 for ease of excitation, low sensory threshold, and aesthetic sensitivity, respectively.

#### Seattle DA classification

The self-reported version of the Seattle DA classification [21, 22] consisting of four categories was used: simple conditioned fear of specific dental stimuli (SI); anxiety about somatic reactions during dental treatment (SII); patients with generalized anxiety states (SIII); distrust of dental personnel (SIV) [21]. The classification was originally used in interviews, but Locker et al. modified it into a self-reported measure [22]. The self-reported version has one item per category with a five-point Likert scale from “Not at all” to “Very much.” The four items correspond to each of the four categories. The items are “I am afraid of things that dentists do such as injections or having my teeth drilled,” “I am afraid of fainting or having a panic attack or a heart attack while having dental treatment,” “In general, I am a nervous person,” and “I am anxious about dental care because I do not trust dentists.”

The scale was translated into Japanese using a structured back-translation process, following core principles of international guidelines for cross-cultural adaptation of self-report measures [41]. First, a Japanese dentist translated the original English version into Japanese. Next, an expert fluent in English and experienced in back-translation retranslated the Japanese version into English. Finally, a third bilingual translator, who was not involved in the earlier steps, compared the original and back-translated versions to review consistency and resolve discrepancies. Since the translated measure was not central to the study’s primary analysis, formal psychometric evaluations of external validity and reliability were not conducted.

#### Exogenous factors

Exogenous factors were measured using the following four questions: ‘Have you ever experienced extremely distressing dental treatment?‘; ‘Have you ever experienced extremely distressing medical treatment?‘; ‘Does your family avoid dental treatment?‘; and ‘Have you ever seen a scary dentist in the media?’ All four questions were answered with ‘Yes’, ‘I don’t know’ or ‘No.’ The authors selected these questions based on previous research and literature [12, 21, 42] .

#### Treatment preferences

Treatment preferences were measured using questions as follows: ‘Do you want to receive dental treatment under general anesthesia?‘, ‘Do you want to receive dental treatment while half asleep with medication?‘, ‘If there was a counseling service to overcome dental anxiety, would you like to attend it?’ Items are rated with a 5-point Likert scale (1 = Not at all willing to undertake it, 5 = Very much willing to undertake it).

### Statistical methods

All statistical analyses were conducted using IBM SPSS (version 27.0). All tests were conducted at a significance level of 0.05. The normality of distribution within variables was tested with the Shapiro-Wilk test. The chi-square test was used to assess differences between categorical variables, and the Mann-Whitney U test was used for ordinal and continuous variables.

Cluster analysis was conducted as a statistical grouping technique to uncover homogenous subgroups (clusters) within a diverse population based on shared characteristics [43]. This unsupervised method is especially useful when group membership is not known in advance and allows for data-driven identification of distinct profiles without pre-defined categories. The two-step cluster analysis was based on eight variables: distressing dental experiences, distressing medical experiences, family dental avoidance, seeing scary dentists from media, fear of pain, pain catastrophizing, and sensory-processing sensitivity. An initial run with an unfixed number of clusters generated candidate solutions (k = 2–5). To select the optimal k, we compared (i) the Bayesian Information Criterion (BIC), (ii) distance-ratio plots, (iii) the average Silhouette Coefficient, and (iv) the max/min cluster-size ratio.

The overall goodness-of-fit of the cluster structure was assessed using the Silhouette Coefficient, < 0.25: no substantial; 0.26–0.50: weak and could be artificial; 0.51–0.70: reasonable; 0.71-1.0: strong [44]. Three χ2 tests or Mann-Whitney U-tests were used to indicate significant differences between identified clusters. When the test indicated a variable was not significant, the cluster analysis was rerun without the variable.

Sensitivity analysis was conducted to test robustness, by repeating Two-Step clustering using only the four Seattle-classification items as predictors. The resulting three-cluster solution was cross-tabulated with the primary two-cluster model, and agreement was quantified with Cohen’s κ. Discrepancies were explored descriptively to determine whether the alternative item set suggested substantively different subtypes.

There are no general guidelines about the sample size necessary for cluster analysis [45]. Formann suggests the minimal sample size includes no less than 2^k^ cases (k = number of variables) [46]. We used a maximum of 7 variables; the minimal sample size is *n* = 128. Thus, we considered our sample size (*N* = 400) was adequate for the cluster analysis. Finally, multivariate logistic regression analysis was used to reveal factors associated with the identified clusters.

## Results

Responses were obtained from 400 participants. One participant was excluded from the analysis because preliminary hierarchical cluster analysis indicated the participant was isolated and had answered all items with the highest or lowest alternative. Data from 399 individuals were used in the analysis. 49.9% of participants were male, 39.3% were employees, and 44.4% had a university degree or higher. The mean age was 43.5 (SD = 13.1) years, and the mean MDAS total score was 18.8 (SD = 5.0).

**Fig. 1:**
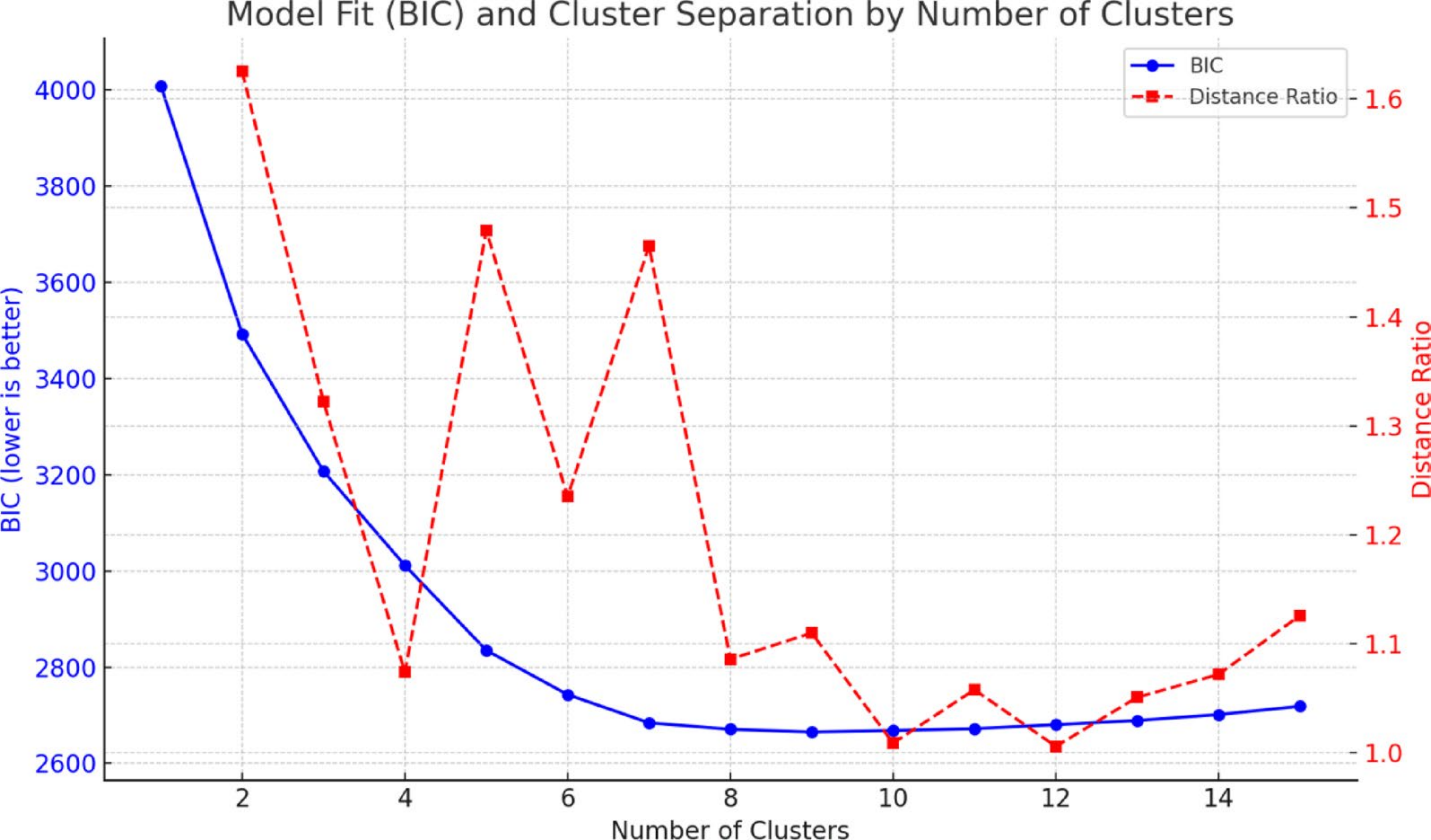
Model fit and cluster separation indices across different numbers of clusters. The graph shows the Bayesian Information Criterion (BIC; blue solid line, left y-axis) and distance ratio (red dashed line, right y-axis) plotted against the number of clusters. Lower BIC values indicate better model fit, whereas higher distance ratios indicate greater separation between clusters. Both indices support the two-cluster solution as optimal: BIC decreases steeply until k = 2–3 and reaches a local minimum around k = 8–10, while the distance ratio shows a marked peak at k = 2. The two-cluster solution was selected for its balance of parsimony and interpretability

Model-fit indices in Fig. 1 supported a two-cluster solution. The Bayesian Information Criterion (BIC) decreased steeply and reached a local minimum at *k = 2*, and the distance ratio peaked at 1.63. The average silhouette for *k = 2* was *0.20*, which indicates weak separation. However, silhouette values remained similarly low (0.20) across *k = 3–5* (see Supplemental Table 1), suggesting that increasing the number of clusters did not meaningfully improve cohesion and separation. Given this and the theoretical interpretability, the two-cluster solution was retained. The cluster size ratio was also acceptable (1.28), indicating a balanced solution.

The first two-cluster solution showed a non-substantial silhouette measure of 0.2 (see Supplemental Tables 2 – first solution). A series of χ² tests or Mann-Whitney U tests indicated that environmental sensitivity and fear of pain were not significantly different among the clusters. The cluster analysis was, therefore, rerun without the two variables. For the final model, the silhouette measure was 0.3, which indicates weak clustering.

**Fig. 2:**
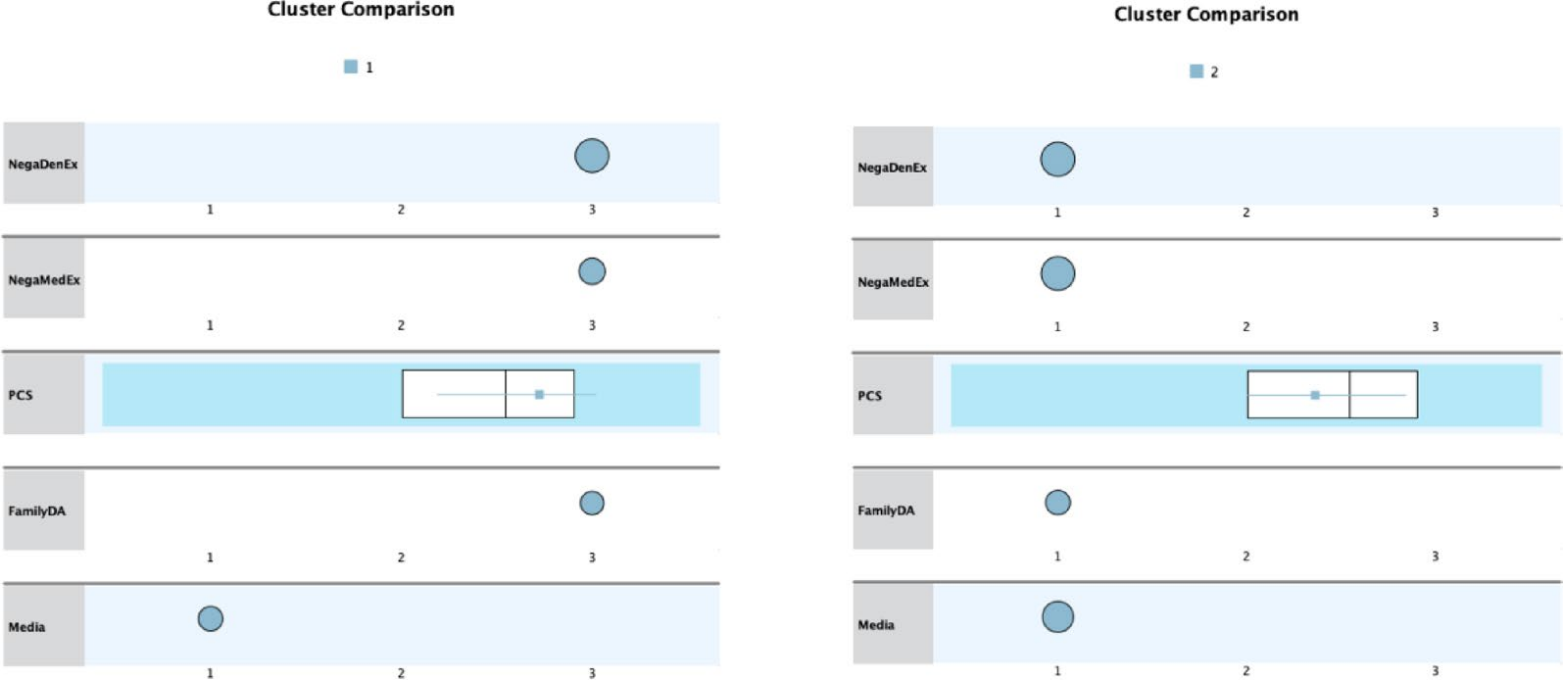
Cluster profiles of individuals with high dental anxiety based on exogenous and endogenous factors. The left and right panels illustrate the profiles of Cluster 1 and Cluster 2, respectively. Each row represents one of the seven clustering variables: two types of past experiences (NegaDenEx: negative dental experience; NegaMedEx: negative medical experience), one continuous psychological measure (PCS: Pain Catastrophizing Scale), and three sources of indirect learning (FamilyDA: dental anxiety in family members; Media: exposure to scary dental scenes in media). For categorical variables (NegaDenEx, NegaMedEx, FamilyDA, Media), response options were coded as 1 = No, 2 = I don’t know, 3 = Yes. For the continuous variable (PCS), boxplots display the distribution within each cluster. The larger circle marker represents the bigger percentages for each categorical item. Compared to Cluster 2, Cluster 1 showed higher frequencies of negative experiences and higher PCS scores, indicating greater vulnerability to both external and internal dental anxiety triggers

Cluster profiles are shown in Fig. 2.

Cluster 1 (C1) (*n* = 173) was a group of participants who reported higher on all exogenous factors; 100% had had a distressing dental experience and participants in this cluster reported higher PCS scores. They also reported significantly higher dental anxiety, more regular dental attendance (24%), and more interest in the treatment of dental anxiety than Cluster 2 (C2). All four types of the Seattle DA classification were significantly more common in C1 than in C2. Cluster 2 (*n* = 226) was characterized by lower levels of exogenous factors than C1, and none of them reported distressing dental experiences but had the lowest rate of regular dental visits (12%) and the highest rate of individuals who had never visited a dentist (14%). For clarity, we refer to C1 as “Trauma & Vulnerability Combined C1 (“Still-Considers”)” and C2 as “Latent-Trait Driven C2 (“Extreme Avoiders”),” respectively. Detailed cluster characteristics are shown in Table 1.

**Table 1:** Differences between dental anxiety clusters based on external and internal factors

	Total *N* = 399			Cluster 1 *N* = 173		Cluster 2 *N* = 226		
	N or mean		% or S.D.	N or mean	% or S.D.	N or mean	% or S.D.	p-value
Gender								0.480
Male	199		49.9%	90	52.0%	109	48.2%	
Female	200		50.1%	83	48.0%	117	51.8%	
Age	43.4		13.12	45.4	12.51	42	13.4	0.003
Education								0.656
High school graduate or lower	155		38.8%	63	36.4%	92	40.7%	
Junior college/Vocational school	71		17.8%	31	17.9%	40	17.7%	
University graduate or above	173		43.4%	79	45.7%	94	41.6%	
MDAS	18.81		4.98	19.86	4.65	18.01	5.08	< 0.001
A-MDAS	7.66		2.17	8.05	2.04	7.35	2.21	0.001
T-MDAS	11.15		3.21	11.80	3.05	10.66	3.25	< 0.001
Distressing dental experiences*								< 0.001
No	184		46.1%	0	0.0%	184	81.4%	
Do not know	42		10.5%	0	0.0%	42	18.6%	
Yes	173		43.4%	173	100.0%	0	0.0%	
Distressing medical experiences*								< 0.001
No	237		59.4%	57	32.9%	180	79.6%	
Do not know	44		11.0%	10	5.8%	34	15.0%	
Yes	118		29.6%	106	61.3%	12	5.3%	
Family dental avoidance*								0.001
No	145		36.3%	50	28.9%	95	42.0%	
Do not know	98		24.6%	38	22.0%	60	26.5%	
Yes	156		39.1%	85	49.1%	71	31.4%	
Seeing scary dentists in media*								0.002
No	246		61.7%	93	53.8%	153	67.7%	
Do not know	71		17.8%	31	17.9%	40	17.7%	
Yes	82		20.6%	49	28.3%	33	14.6%	
FPQ	32.30		7.95	32.51	7.91	32.14	8.00	0.771
PCS*	47.11		10.74	49.38	10.11	45.38	10.90	< 0.001
HSPS	4.64		1.10	4.78	1.02	4.54	1.15	0.090
Dental visiting behavior								0.002
Regularly	69		17.3%	42	24.3%	27	11.9%	
Irregularly	218		54.6%	95	54.9%	123	54.4%	
Do not go even if in pain.	80		20.1%	36	20.8%	44	19.5%	
Never been to a dentist	32		8.0%	0	0.0%	32	14.2%	
Self-reported oral health								0.002
Average/bad/very bad	344		86.2%	160	92.5%	184	81.4%	
Very good/good	55		13.8%	13	7.5%	42	18.6%	
Seattle Ⅰ (Simple dental phobia)								0.020
Not at all/a little/somewhat	146		36.6%	53	30.6%	93	41.2%	
Much/ very much	253		63.4%	120	69.4%	133	58.8%	
Seattle Ⅱ (Anxiety about somatic reactions)								0.011
Not at all/ a little/ somewhat	283		70.9%	111	64.2%	172	76.1%	
Much/ very much	116		29.1%	62	35.8%	54	23.9%	
Seattle Ⅲ (Being nervous generally)								0.018
Not at all/ a little/ somewhat	221		55.4%	85	49.1%	136	60.2%	
Much/ very much	178		44.6%	88	50.9%	90	39.8%	
Seattle Ⅳ (Distrust of dental personnel)								< 0.001
Not at all/ a little/ somewhat	270		67.7%	99	57.2%	171	75.7%	
Much/ very much	129		32.3%	74	42.8%	55	24.3%	
Interests for GA								0.004
Not at all/ a little/ somewhat	245		61.4%	92	53.2%	153	67.7%	
Much/ very much	154		38.6%	81	46.8%	73	32.3%	
Interests for IVS								0.014
Not at all/ a little/ somewhat	236		59.1%	90	52.0%	146	64.6%	
Much/ very much	163		40.9%	83	48.0%	80	35.4%	
Interests for CBT								0.063
Not at all/ a little/ somewhat	241		60.4%	95	54.9%	146	64.6%	
Much/ very much	158		39.6%	78	45.1%	80	35.4%	

* Using this two-factor cluster analysis. P value for Chi-square test or Mann-Whitney test.

*MDAS* Modified Dental Anxiety Scale, *FPQ* Fear of Pain Questionnaire, *PCS* Pain Catastrophizing Scale, *HSPS* Highly Sensitive Person, *GA* General anesthesia, *IVS* Intravenous Sedation, *CBT* Cognitive Behavior Treatment.

As a sensitivity analysis, a two-step cluster analysis was conducted using the four items of the Seattle DA classification. This analysis yielded a three-cluster solution, which was supported by model fit indices. However, the average silhouette coefficient was low (0.20), indicating weak cohesion and separation. Notably, all four items moved in the same direction across clusters, suggesting a common underlying structure. Details of the clusters are presented in Supplemental Table 3. Among the three psychological variables tested (FPQ, HSPS, and PCS), only PCS showed significant between-cluster differences. Cluster 2 C2: Latent-Trait Driven had the highest proportion of individuals endorsing all four Seattle DA classifications.

Logistic regression analysis revealed that less irregular dental attendance, worse self-reported oral health, greater anxiety about somatic reactions (Seattle class SII), greater distrust of dental personnel (Seattle class SIV), and greater interest in general anesthesia were more often reported in C1: Trauma & Catastrophizing Compounded than in C2: Latent-Trait Driven (Table 2).

**Table 2:** The results of multivariate logistic regression analysis for cluster 1 vs. cluster 2

	B	*p*-value	Lower 95% CI	Exp (B)	Upper 95% CI
Gender	− 0.076	0.748	0.584	0.927	1.471
Age	0.018	0.04	1.001	1.018	1.036
Irregular Dental Attendance	− 0.876	< 0.001	0.299	0.416	0.580
Self-reported good oral health	− 0.449	< 0.001	0.504	0.639	0.808
Seattle Ⅰ (Simple dental phobia)	0.135	0.212	0.926	1.144	1.413
Seattle Ⅱ (Anxiety about somatic reactions)	0.181	0.046	1.003	1.199	1.433
Seattle Ⅲ (Being nervous generally)	− 0.031	0.767	0.792	0.97	1.188
Seattle Ⅳ (Distrust of dental personnel)	0.265	0.011	1.062	1.303	1.599
Interests for GA	0.273	0.041	1.012	1.313	1.704
Interests for IVS	− 0.034	0.815	0.727	0.967	1.285
Interests for CBT	0.030	0.778	0.836	1.031	1.271

*GA* General anesthesia, *IVS* Intravenous Sedation, *CBT* Cognitive Behavior Treatment.

Independent: cluster (reference: cluster 2)

## Discussion

This study identified two empirically derived subtypes among adults with high dental anxiety, using a person-centered clustering approach based on both exogenous (learning/experiential) and endogenous (psychological/biological vulnerability) factors. The clusters—“Trauma & Catastrophizing Compounded” (C1 / Still-Considers) and “Latent-Trait Driven” (C2 / Extreme Avoiders)—demonstrated distinct dental distrust, pain/sensory sensitivity, self-reported oral health, patterns of dental attendance, and treatment preference.

Trauma & Catastrophizing Compounded C1 was characterized by universal reports of highly distressing dental experiences, elevated pain catastrophizing (PCS), and greater distrust toward dental professionals. Despite these burdens, 24% of this group attended dental care regularly and expressed interest in treatments for dental anxiety. This profile suggests a layered pattern in which internal vulnerabilities are compounded by external trauma—individuals who remain motivated to seek care despite high fear. They can be called “Still-Considers”.

Latent-Trait Driven C2 showed no traumatic dental experiences and low scores on exogenous factors, yet included the highest proportion (14%) of individuals who had never visited a dentist. Their elevated FPQ and HSPS scores suggest a trait-based vulnerability rooted in pain-related fear and sensory sensitivity. In contrast to Trauma & Catastrophizing Compounded C1, their anxiety appears internally driven and behaviorally avoidant from the outset. We describe this group as “Extreme Avoiders.”

While van Houtem et al.‘s three-factor model—comprising fear of invasive treatment, loss of control, and physical sensations [23]—has contributed to understanding dimensions of dental fear, it differs in focus from our clustering approach. Their framework centers on fear content (i.e., what patients fear), whereas these model focuses on the possible etiological origins—traumatic experiences and constitutional vulnerability. The Seattle DA classification-based sensitivity analysis, which included the four dental fear types, showed poor concordance with our two-cluster solution (Cohen’s κ = − 0.092). This discrepancy suggests that category-driven models and cause-based clustering may capture distinct layers of heterogeneity in dental anxiety.

This etiological focus provides added clinical value, as it aligns more directly with mechanisms that can be targeted through intervention. For instance, the Trauma × High PCS profile in Trauma & Catastrophizing Compounded C1 resembles the mutual maintenance model described in PTSD and chronic pain, wherein intrusive memories and cognitive catastrophizing mutually reinforce distress [47]. This supports the use of graded exposure to dental procedures and cognitive restructuring in CBT for this group [48].

Notably, the Trauma & Catastrophizing Compounded cluster also showed high distrust toward dental professionals (Seattle SIV). This aligns with findings from Yuan et al., who demonstrated in a person-centered model that lower trust predicts higher dental anxiety [49]. Their scoping review further noted that although patient-centered communication is recognized as critical in building trust, empirical research on how trust in dental professionals is formed remains scarce [50]. These findings reinforce that rebuilding trust must be a core element of intervention for individuals in the trauma-related cluster.

Although neither FPQ nor HSPS differed significantly between clusters, both Trauma & Catastrophizing Compounded C1 and Latent-Trait Driven C2 scored well above Japanese norms (FPQ mean = 28.7, SD = 7.4; HSP mean = 4.1, SD = 1.1) [34, 40], with z-scores of approximately + 0.49. This suggests that elevated pain-related fear and sensory sensitivity are shared internal vulnerabilities across both clusters among those with high dental anxiety. Notably, prior studies have linked both FPQ and HSPS to genetic and temperamental traits, supporting the view that they may represent stable, trait-level predispositions [17, 51–53]. The Trauma & Catastrophizing Compounded C1 then adds a layer of traumatic dental experiences and high catastrophizing (PCS), resulting in a more complex anxiety profile. These individuals may benefit from interventions combining cognitive restructuring and trauma-informed care.

In contrast, the Latent-Trait Driven C2 demonstrates that internal vulnerability alone, even in the absence of trauma, can lead to marked avoidance, with 14% never having visited a dentist. Given their low external learning signals but elevated internal susceptibility, environmental adaptations (e.g., noise reduction, dimmed lighting) [54] and emotional regulation strategies may be particularly helpful for this group. Their low motivation for treatment of DA also highlights the need for early-stage engagement strategies, such as motivational interviewing and preventive outreach. This two-stage model—shared vulnerability, with or without trauma overlay—may clarify why some anxious individuals remain engaged (“Still-Considers”) while others withdraw silently from care (Table 3).

**Table 3:** Cluster-specific characteristics and intervention strategies

Item	Cluster 1	Cluster 2
Label (Interpretation)	Trauma & Catastrophizing Compounded (“Still-Considers”)	Latent-Trait Driven (“Extreme Avoiders”)
Key Characteristics	- Distressing dental experience: 100% - Higher PCS Elevated HSPS (z = 0.62) and FPQ (z = 0.52) vs. general population − 24% regular attendance - High distrust (Seattle IV)	- No distressing dental experience - Lower PCS Elevated HSPS (z = 0.46) and FPQ (z = 0.40) vs. general population − 14% never visited - Lowest motivation for treatment
	Layered high dental anxiety	Trait-Driven high dental anxiety
Psychological Interpretation	Trait vulnerability + traumatic learning → More intense, but some motivation remains	No trauma; internal vulnerability only → Silent avoidance, low visibility in care
Suggested Clinical Approaches	- Graded exposure - Cognitive restructuring - Trust-building communication - Continuity of care	- Environmental adaptations (noise/light) - Early engagement in risk awareness - Motivational interviewing - Gentle, non-invasive first contact

*FPQ* Fear of Pain Questionnaire, *PCS* Pain Catastrophizing Scale, *HSPS* Highly Sensitive Person Scale.

Despite expectations, the separation between clusters was modest, as evidenced by low silhouette coefficients and sensitivity analyses using the Seattle DA classification. This suggests that as dental anxiety increases in level, dimensional distinctions may collapse into a unified high-fear complex. Prior large-scale population studies have similarly found that most highly anxious individuals report multiple overlapping fears [23]. The discrepancy in subgroup membership between the factor-based and Seattle DA classification-based clustering further supports the continuum hypothesis of dental anxiety. From a clinical standpoint, this reinforces the need for tailored strategies: for low-to-moderate anxiety cases, factor-specific approaches (e.g., pain management versus control enhancement) may suffice. On the other hand, high-anxiety individuals likely require more integrative, multi-component interventions including exposure, cognitive restructuring, and physiological regulation.

This study applied a person-centered clustering approach based on both exogenous and endogenous factors to identify subtypes of high dental anxiety. This framework may inform the development of tailored interventions that address individual needs. The study also used well-validated psychological measures and included a demographically balanced sample (*N* = 399), enhancing the reliability and relevance of the findings. However, some limitations should be noted. The silhouette coefficient (0.30) indicates weak cluster separation, suggesting that more comprehensive models incorporating variables such as temperament, personality, alexithymia, or self-efficacy may yield clearer subtypes. Second, the reliance on self-reporting via an online panel introduces the possibility of social desirability bias. Future studies should incorporate clinician-rated and observational data. Third, the sample was limited to Japanese adults, and the cultural generalizability of findings remains to be tested.

Future studies should expand on this work by conducting latent class analyses with broader variable sets, developing and evaluating cluster-specific CBT protocols and environmental support packages, and testing trust-enhancing communication strategies. International comparative research is also needed to examine how culture influences the distribution and expression of dental anxiety subtypes.

Taken together, these findings support a novel, empirically derived typology of high dental anxiety, comprising (1) a Trauma & Catastrophizing type and (2) a Latent-Trait Driven type. This person-centered framework may inform future clinical assessment and guide the development of tailored intervention strategies.

## Conclusion

This study offers novel insight into the heterogeneity of high dental anxiety by identifying two empirically derived subtypes. While previous models have described dimensions of fear, this framework distinguishes patient groups based on their etiological profiles—external trauma, internal vulnerability, or both. These findings may inform more precise, subtype-matched interventions and ultimately improve access to care for underserved populations.

## Supplementary Information

Supplementary material 1

## Data Availability

The datasets used and/or analysed during the current study are available from the corresponding author on reasonable request.

## Abbreviations

MDAS: Modified dental anxiety scale
FPQ: Fear of pain questionnaire
PCS: Pain catastrophizing scale
HSPS: Highly sensitive person scale
GA: general anesthesia
IVS: Intravenous sedation
CBT: Cognitive behavior treatment
