# Supplementary material for: Exploring high dental anxiety subtypes using cluster analysis approach: a cross-sectional study
Source: BMC Oral Health. 2025 Oct 1;25:1506. doi: 10.1186/s12903-025-06820-7 (PMC12486690; doi:10.1186/s12903-025-06820-7)
Supplement: Supplementary file 1 — Supplementary material 1 [file 12903_2025_6820_MOESM1_ESM.docx]

| Supplemental 1. The assessments of cluster numbers | | | | |
| --- | --- | --- | --- | --- |
| The number of factors | Two | Three | Four | Five |
| Cluster 1 | 224 (56.1%) | 121 (30.3%) | 121 (30.3%) | 121 (30.3%) |
| Cluster 2 | 175 (43.9%) | 113 (28.3%) | 118 (29.6%) | 69 (17.3%) |
| Cluster 3 | – | 165 (41.4%) | 95 (23.8%) | 95 (23.8%) |
| Cluster 4 | – | – | 65 (16.3%) | 49 (12.3%) |
| Cluster 5 | – | – | – | 65 (16.3%) |
| Max/Min cluster ratio | 1.28 | 1.46 | 1.86 | 2.47 |
| Average silhouette | 0.2 | 0.2 | 0.2 | 0.2 |

| Supplemental 2. Contribution of selected exogenous and endogenous variables to the cluster formation | | | | | | | | | |
| --- | --- | --- | --- | --- | --- | --- | --- | --- | --- |
|  | First solution | | | Second solution | | | Final solution | | |
| Variable | PImp | χ2 or Z value | p-value | PImp | χ2 or Z value | p-value | PImp | χ2 or Z value | p-value |
| Distressing dental experiences | 1.00 | 314.767 | <0.001 | 1.00 | 353.49 | <0.001 | 1.00 | 399.00 | <0.001 |
| Distressing medical experiences | 0.70 | 221.340 | <0.001 | 0.55 | 193.84 | <0.001 | 0.37 | 147.37 | <0.001 |
| PCS | 0.08 | 4.895 | <0.001 | 0.06 | 4.347 | <0.001 | 0.04 | -3.761 | <0.001 |
| Seeing scary dentists in the media | 0.06 | 18.193 | <0.001 | 0.04 | 14.08 | <0.001 | 0.03 | 12.07 | 0.002 |
| Family dental care avoidance | 0.05 | 16.444 | <0.001 | 0.04 | 13.25 | 0.001 | 0.03 | 13.36 | 0.001 |
| HSPS | 0.04 | 2.699 | 0.007 | 0.02 | 1.921 | 0.550 | – | – | – |
| FPQ | 0.01 | 1.352 | 0.176 | – | – | – | – | – | – |
|  | Cluster 1 = 224 (56.1%) | | | Cluster 1 = 214 (53.6%) | | | Cluster 1 = 173 (43.4%) | | |
|  | Cluster 2 = 175 (43.9%) | | | Cluster 2 = 185 (46.4%) | | | Cluster 2 = 226 (56.6%) | | |
|  | **Max/Min cluster ratio** = 1.28 | | | **Max/Min cluster ratio** = 1.16 | | | **Max/Min cluster ratio** = 1.31 | | |
|  | Average silhouette = 0.20 | | | Average silhouette = 0.30 | | | Average silhouette = 0.30 | | |
| PImp, predictor importance  FPQ= Fear of Pain Questionnaire; PCS= Pain Catastrophizing Scale; HSPS= Highly Sensitive Person Scale | | | | | | | | | |

=

| Supplemental table 3. Differences between dental anxiety clusters based on four items of the Seattle DA classification. | | | | | | | |
| --- | --- | --- | --- | --- | --- | --- | --- |
|  | Cluster 1 N=130 | | Cluster 2 N = 123 | | Cluster 3 N = 146 | |  |
|  | N or mean | % or S.D. | N or mean | % or S.D. | N or mean | % or S.D. | p-value |
| Gender |  |  |  |  |  |  | 0.277 |
| Male | 72 | 55.4% | 60 | 48.8% | 67 | 45.9% |  |
| Female | 58 | 44.6% | 63 | 51.2% | 79 | 54.1% |  |
| Age | 43.1 | 13.7 | 43.5 | 12.3 | 43.7 | 13.3 | 0.516 |
| MDAS | 17.9 | 4.0 | 23.0 | 2.8 | 16.1 | 5.0 | <0.001 |
| A-MDAS | 7.2 | 1.8 | 9.2 | 1.5 | 6.8 | 2.3 | <0.001 |
| T-MDAS | 10.7 | 2.5 | 13.8 | 1.8 | 9.4 | 3.3 | <0.001 |
| Negative dental experiences |  |  |  |  |  |  | <0.001 |
| Yes (1) | 52 | 40.0% | 74 | 60.2% | 47 | 32.2% |  |
| No/ I don’t know (2) | 78 | 60.0% | 49 | 39.8% | 99 | 67.8% |  |
| Negative medical experiences |  |  |  |  |  |  | <0.001 |
| Yes (1) | 37 | 28.5% | 52 | 42.3% | 29 | 19.9% |  |
| No/ I don’t know (2) | 93 | 71.5% | 71 | 57.7% | 117 | 80.1% |  |
| Family dental anxiety |  |  |  |  |  |  | 0.666 |
| Yes (1) | 52 | 40.0% | 51 | 41.5% | 53 | 36.3% |  |
| No/ I don’t know (2) | 78 | 60.0% | 72 | 58.5% | 93 | 63.7% |  |
| Seeing fearful dentists in the media |  |  |  |  |  |  | 0.007 |
| Yes (1) | 26 | 20.0% | 36 | 29.3% | 20 | 13.7% |  |
| No/ I don’t know (2) | 104 | 80.0% | 87 | 70.7% | 126 | 86.3% |  |
| FPQ | 31.8 | 6.8 | 35.0 | 8.3 | 30.5 | 8.1 | <0.001 |
| PCS | 45.7 | 8.6 | 52.6 | 10.5 | 43.7 | 10.9 | <0.001 |
| HSP | 45.4 | 9.2 | 51.9 | 10.4 | 42.9 | 11.4 | <0.001 |
| Behavior |  |  |  |  |  |  | 0.072 |
| regularly (1) | 17 | 13.1% | 29 | 23.6% | 23 | 15.8% |  |
| irregularly/never (2) | 113 | 86.9% | 94 | 76.4% | 123 | 84.2% |  |
| Self-reported oral health |  |  |  |  |  |  | 0.584 |
| Average/bad/ very bad (1) | 112 | 86.2% | 109 | 88.6% | 123 | 84.2% |  |
| Very good/ good/ (2) | 18 | 13.8% | 14 | 11.4% | 23 | 15.8% |  |
| Seattle Ⅰ* (Simple dental phobia) |  |  |  |  |  |  | <0.001 |
| Not at all/ a little/ somewhat | 58 | 44.6% | 9 | 7.3% | 79 | 54.1% |  |
| Much/ very much | 72 | 55.4% | 114 | 92.7% | 67 | 45.9% |  |
| Seattle Ⅱ* (Anxiety about somatic reactions) | |  |  |  |  |  | <0.001 |
| Not at all/ a little/ somewhat | 89 | 68.5% | 53 | 43.1% | 141 | 96.6% |  |
| Much/ very much | 41 | 31.5% | 70 | 56.9% | 5 | 3.4% |  |
| Seattle Ⅲ* (Being nervous generally) |  |  |  |  |  |  | <0.001 |
| Not at all/ a little/ somewhat | 76 | 58.5% | 23 | 18.7% | 122 | 83.6% |  |
| Much/ very much | 54 | 41.5% | 100 | 81.3% | 24 | 16.4% |  |
| Seattle Ⅳ* (Distrust of dental personnel) |  |  |  |  |  |  | <0.001 |
| Not at all/ a little/ somewhat | 85 | 65.4% | 41 | 33.3% | 144 | 98.6% |  |
| Much/ very much | 45 | 34.6% | 82 | 66.7% | 2 | 1.4% |  |
| GA |  |  |  |  |  |  | 0.097 |
| Not at all/ a little/ somewhat | 86 | 66.2% | 66 | 53.7% | 93 | 63.7% |  |
| Much/ very much | 44 | 33.8% | 57 | 46.3% | 53 | 36.3% |  |
| IVS |  |  |  |  |  |  | 0.316 |
| Not at all/ a little/ somewhat | 83 | 63.8% | 67 | 54.5% | 86 | 58.9% |  |
| Much/ very much | 47 | 36.2% | 56 | 45.5% | 60 | 41.1% |  |
| CBT |  |  |  |  |  |  | 0.204 |
| Not at all/ a little/ somewhat (1) | 85 | 65.4% | 67 | 54.5% | 89 | 61.0% |  |
| Much/ very much (2) | 45 | 34.6% | 56 | 45.5% | 57 | 39.0% |  |
| * Using this two-factor cluster analysis. P value for Chi-square test or Mann-Whitney test. | | | | | | | |
| MDAS = Modified Dental Anxiety Scale; FPQ= Fear of Pain Questionnaire; PCS= Pain Catastrophizing Scale; HSPS= Highly Sensitive Person; GA = general anesthesia; IVS = Intravenous Sedation; CBT = Cognitive Behavior Treatment. | | | | | | | |

| **Supplemental Table 4. Cross-tabulation of cluster solutions based on exogenous/endogenous variables and Seattle DA classification items** | | | | |
| --- | --- | --- | --- | --- |
| **Cluster (Exogenous/Endogenous)** | **Seattle Cluster 1** | **Seattle Cluster 2** | **Seattle Cluster 3** | **Total** |
| Cluster 1 | 52 (40.0%) | 74 (60.2%) | 47 (32.2%) | 173 (43.4%) |
| Cluster 2 | 78 (60.0%) | 49 (39.8%) | 99 (67.8%) | 226 (56.6%) |
| **Total** | 130 (100.0%) | 123 (100.0%) | 146 (100.0%) | 399 (100.0%) |
